# Editorial: Advancements in analytical chemistry

**DOI:** 10.3389/fchem.2026.1961888

**Published:** 2026-08-24

**Authors:** Huangxian Ju, Serge Cosnier

**Affiliations:** 1 State Key Laboratory of Analytical Chemistry for Life Science, School of Chemistry, Nanjing University, Nanjing, China; 2 Department of Chemistry, University Grenoble Alpes, Grenoble, France

**Keywords:** analytical chemistry, analytical methodologies, detection instruments, highlights of contributions, measurement science

## 1 Introduction

Analytical chemistry is a measurement science that provides data on the chemical composition, structure, and concentrations of substances, their spatiotemporal distribution, and their interactions in different states, including evolutionary information. The discipline aims to propose new measurement principles, design various analysis strategies and methods, and develop various detection devices, measurement instruments, and related software to probe the spatio-temporal variation rules of material composition and properties (Ju, 2013). A defining characteristic of this discipline is the use of interactions between matter and various force fields, in addition to new research achievements from different disciplines, to maximize the acquisition of necessary information and relevant scientific data. Analytical chemistry plays an important role in overcoming diseases, promoting health, controlling and protecting the environment, developing new energy resources, improving the quality of human life, and enhancing the level of information transmission and processing. Developed analytical methods, detection technologies, and instruments are extensively applied in different fields, including disease diagnosis, new drug development, environmental monitoring, studying life processes, ensuring food safety, controlling product quality, space exploration, forensic medicine, and even anti-terrorism. As a Frontier discipline, analytical chemistry provides important support for the development of life science, materials science, energy science, environmental science, and space science.

Currently, analytical chemistry is integrating multidisciplinary research achievements, including artificial intelligence (AI) technology, nanotechnology, and single-atom catalysis, to develop new theoretical frameworks and new Frontier research fields at the fundamental level. The development of other related disciplines and the progress of human society constantly challenge analytical chemistry, promoting the continuous advancement of analytical methodologies and detection instruments. This Research Topic, “*Advancements in Analytical Chemistry,*” spotlights recent developments in analytical methodology to promote interdisciplinary collaboration, integration, and the expansion of analytical chemistry applications in new, cutting-edge fields. This Research Topic features five review articles and five original research articles on innovative analytical and bioanalytical chemistry methods, emphasizing advancements that address critical challenges in different areas such as biomedicine, the environment, forensics, and diagnostics.

## 2 Highlights of contributions to the Research Topic

The first contribution to this Research Topic constructed a hybrid enhanced Raman scattering probe, which was made by assembling a layer of graphene and silver nanoparticles on a hexagonal ZnO microrod for the ultrasensitive analysis of pathogens (Shan et al.). This study highlights a label-free approach for a variety of clinically relevant biomolecules, even within single cells, to achieve the early diagnosis of disease.

In the second contribution, a gelatin-based fixation method was developed to obtain intact histological sections suitable for mass spectrometric imaging (MSI) analysis. This method used MALDI-FT-ICR MSI to investigate whole-body lipid and metabolite distribution in *S. nobilis* (Redureau et al.). This method paves the way for broader applications of spatial lipidomics in other small biosystems and animals, particularly those previously inaccessible to detailed biochemical analysis.

Pu and Wu’s contribution reviewed developments in nucleic acid probe-based techniques for detecting gene point mutations, with an emphasis on strategies involving pure nucleic acid probes and the synergistic use of enzymes, nucleic acid analogs, and nanotechnology (Pu and Wu). This article summarized the principles, advantages, and limitations of these technologies, in addition to exploring the application of AI technology in nucleic acid probes and future challenges in the field.

Shen et al. used gas chromatography–ion mobility spectrometry (GC-IMS) combined with chemometrics to analyze the differences in volatile organic compounds (VOCs) in the serum of patients with and without hepatocellular carcinoma (HCC). This analysis explored new HCC biomarkers (Shen et al.), demonstrating that VOC detection can be used as an auxiliary diagnostic tool or for population-wide screening.

The fifth article introduced types of dissolved gas analysis and milestone research progress for online detection from 2019 to 2025, including online gas chromatographic analysis systems, spectral analysis, and gas sensor methods (Mao et al.). The article presented the development trend of promoting intelligent and accurate power equipment status monitoring.

Chen et al. systematically summarized recent advances in label-free surface-enhanced Raman scattering (SERS)-based cancer diagnostics (Chen et al.). It outlined the fundamental principles of SERS, key substrate fabrication methodologies, and essential spectral analysis techniques, highlighting applications of this technology in liquid biopsy, and discussing current challenges and future directions in this rapidly evolving field.

The seventh contribution, from UAE University, used attenuated total reflectance Fourier transform infrared (ATR-FT-IR) spectroscopy, combined with multivariate chemometric models, to classify 69 real-world gunpowder samples (Aljanaahi et al.). The study demonstrated that both the selection and sequence of spectral preprocessing steps significantly influence model performance. This article provided a rapid, non-destructive, and interpretable screening approach for forensic laboratories.

The study by Qin et al. used a gradient elution method to establish reverse-phase high-performance liquid chromatography (RP-HPLC) for the separation of resmetirom and its related substances (Qin et al.). This method is simple to operate and yields dependable results, providing a practical and efficient analytical tool for the quality control of resmetirom and its formulations, along with the identification of new degradation products.

Jofre et al. evaluated an alternative quantitative strategy, multi-isotope calibration (MICal), for multi-elemental determination by inductively coupled plasma mass spectrometry (ICP-MS) in complex agro-food matrices (Jofre et al.). This method was successfully applied to determine Cd, Cu, Mo, Pb, V, and Zn in real samples, demonstrating that integrating MICal with infrared-assisted digestion provides a robust, sustainable, and practically efficient strategy for routine multielemental analysis.

The final review article, by Eliw et al., summarized the current knowledge on specimen selection and the factors influencing drug absorption, distribution, and elimination. It also highlighted analytical strategies ranging from chromatographic and mass spectrometric methods to emerging electrochemical, optical, and biosensor platforms (Eliw et al.). The study critically evaluated advances in sample preparation, miniaturization, and portable analytical devices in terms of sensitivity, specificity, and applicability to forensic and clinical settings.
